# Solid ameloblastomas -Retrospective clinical and histopathologic study of 54 cases

**DOI:** 10.1590/S1808-86942010000200005

**Published:** 2015-10-19

**Authors:** Giovana Medeiros Fulco, Cassiano Francisco Weege Nonaka, Lélia Batista de Souza, Márcia Cristina da Costa Miguel, Leão Pereira Pinto

**Affiliations:** Dentistry student, scientific initiation scholarship holder - Federal University of Rio Grande do Norte; MSc in Oral Pathology, PhD student in oral pathology - Federal University of Rio Grande do Norte; PhD in Oral Pathology - Professor of the Graduate Program in Oral Pathology - Federal University of Rio Grande do Norte; PhD in Oral Pathology, Professor of the Graduate Program in Oral Pathology of the Federal University of Rio Grande do Norte; PhD in Oral Pathology, Professor of the Graduate Program in Oral Pathology - Federal University of Rio Grande do Norte

**Keywords:** ameloblastoma, mandible, maxilla, odontogenic tumors

## Abstract

Recently, the World Health Organization (WHO) excluded the desmoplastic pattern from the histopathological spectrum of solid ameloblastomas and classified it as a distinct variant, named desmoplastic ameloblastoma.

**Aim:**

To perform a retrospective analysis of the clinicopathologic aspects in a case series of solid ameloblastomas.

**Study design:**

Cross-sectional cohort study.

**Materials and methods:**

Data regarding age, gender, location and clinical characteristics were retrieved from patient records. Histological sections were evaluated regarding existing histological patterns and the predominant histological pattern. Cases were classified according to the study of Waldron and El-Mofty (1987) and the WHO classification of 2005.

**Results:**

A total of 54 cases were identified, with similar gender distribution and a mean age of 38.3 years. Fifty three cases (98.1%) affected the mandible. Forty nine cases (90.8%) were classified as solid ameloblastomas, 3 (5.6%) as desmoplastic ameloblastomas, and 2 (3.7%) as hybrid lesions. The most frequent histological patterns in solid ameloblastomas were follicular (77.6%), acanthomatous (69.4%), and plexiform (65.3%). Focal areas of desmoplastic ameloblastomas were identified in 11 solid ameloblastomas (22.4%).

**Conclusion:**

Despite its characterization as a distinct variant, our results revealed that focal areas of desmoplastic ameloblastomas can be observed with some frequency in conventional ameloblastomas.

## INTRODUCTION

Ameloblastoma is a benign epithelial odontogenic tumor, locally invasive and of slow growth^1^. Numerous histological patterns can be seen in these lesions, such as: follicular, plexiform, acanthomatous, desmoplastic, basal cells and granular^2^. Up to 1992, the World Health Organization (OMS) recognized the existence of 3 distinctive clinicopathologic variants of ameloblastoma, called conventional solid ameloblastoma, unicystic ameloblastoma and peripheral ameloblastoma^3^.

Case reports and retrospective studies^4^, ^5^, ^6^, ^7^, ^8^ carried out after the WHO's 1992 classification^3^ reported important clinical and image differences between ameloblastomas comprised exclusively of the desmoplastic pattern and solid lesions made by the remaining histological patterns. Thus, in their most recent classification of odontogenic tumors, published in 2005, the WHO excluded the desmoplastic pattern from the histological spectrum of solid ameloblastomas and placed it as a distinctive variant called desmoplastic ameloblastoma^9^.

According to the WHO^9^, ameloblastomas which have both solid and desmoplastic areas are called hybrid lesions. It is suggested that the hybrid lesions represent a coalition of tumors^10^^,^^11^. Notwithstanding, Melrose^12^ states that the word “hybrid” does not have a clearly defined purpose and, if considered literally, can overestimate the meaning of seeing areas of desmoplastic ameloblastoma in combination with islets of solid ameloblastoma.

Having the recent classification of odontogenic tumors from the WHO^9^, the present study aims at doing a retrospective analysis of the clinical and histopathological findings from a number of solid ameloblastomas filed in the Laboratory of Oral Pathology of the Department of Dentistry of the Federal University of Rio Grande do Norte (UFRN).

## MATERIALS AND METHODS

We carried out a retrospective study in a series of cases of solid ameloblastomas using clinical charts and histology slides found in the files of the Laboratory of Oral Pathology of the Department of Dentistry of the UFRN. This study was approved by the Ethics in Research Committee of the UFRN (Document # 171/2008).

We used 54 cases of solid ameloblastomas. The criteria established in order to include the cases in the sample were the presence of a recorded chart, with the gender of the patient and anatomical location of the lesions, as well as the existence of enough biological material in the paraffin blocks in order to prepare the histology slides. Any case which did not match the criteria previously established was taken off the study.

For the clinical study, we collected data regarding the patients' gender and age, as well as anatomical location, symptoms and time of lesion development.

For the morphological study we used 5mm thick slides cut from the paraffin material, dyed by the hematoxylin-eosin technique. The specimens were analyzed under light microscopy (Olympus X31 microscope), identifying the histological patterns present and the predominant histological pattern in the lesions. Later on, having as reference the study led by Waldron and El-Mofty^13^ and the classification of odontogenic tumors by the WHO^9^, The cases were classified into desmoplastic solid ameloblastomas or hybrid lesions. The criteria used to classify the cases are presented on Chart 1.

**Chart 1:** Criteria used to classify the cases of solid ameloblastomas, desmoplastic ameloblastomas and hybrid lesions.

**Solid ameloblastoma** • Lesions made up by one or more of the following histological patterns: follicular, acanthomatous, plexiform, basal cells, granular cells. • Lesions made up by focal areas of the desmoplastic pattern in association to large masses made up by one or more of the following histological patterns: follicular, acanthomatous, plexiform, basal cells, granular cells.
**Desmoplastic ameloblastoma** • Lesions made up exclusively by the desmoplastic pattern. • Lesions made up by the desmoplastic pattern associated with rare islets of similar aspect to that of the follicular one found in solid ameloblastomas.
**Hybrid lesion** • Lesions made up by masses of desmoplastic ameloblastoma associated to significant areas of solid ameloblastoma.

The data obtained was plotted on electronic spreadsheets using Microsoft Excel (Microsoft Corporation), and later on exported to the Statistical Package for Social Sciences (SPSS 13.0), from which we obtained the mean values, absolute and percentage frequencies through descriptive statistics techniques. In order to analyze the differences between solid ameloblastomas, desmoplastic ameloblastomas and hybrid lesions in relation to gender, anatomical location, region and symptoms, we used the Chi-Squared test, considering p<0.05 as significant value.

## RESULTS

The analysis of the clinical data revealed a similar involvement between the genders, with 27 cases (50.0%) diagnosed in men and 27 (50.0%) in women. The age of the patients varied between 12 and 92 years, with a mean value upon diagnosis of 38.3 years. Men and women revealed mean ages of 38.2 and 38.4 years, respectively. As far as anatomical location is concerned, we noticed a predilection for the mandible (98.1%). Most of the cases were asymptomatic (82.1%) and were located in the posterior portion of the gnathic bones (66.7%). Time of evolution varied between 1 month and 33 years, with a mean value of 34.9 months. The diameter of the lesions varied between 0.7 cm and 15.0 cm, with a mean value of 4.2 cm.

After histopathological evaluation, 49 cases (90.8%) were classified as solid ameloblastomas, 3 (5.6%) as desmoplastic ameloblastomas and 2 (3.7%) as hybrid lesions. The clinical data related to gender, anatomical location, region and symptoms concerning each type of ameloblastoma are presented on Table 1. We did not notice statistically significant differences between solid ameloblastomas, desmoplastic ameloblastomas and hybrid lesions as far as gender is concerned (p = 0.838), anatomical location (p = 0.949), region (p = 0.091) and symptoms (p = 0.358).

**Table 1:** Distribution of ameloblastomas according to gender, anatomical localization, region and symptoms. Natal - RN, 2009.

Type of ameloblastoma							
			Desmoplastic	Hybrid lesion			
Variable	Category	Solid n (%)	n (%)	n (%)	Total n (%)	X2	p
Gender	Male	25 (51,0%)	1 (33,3%)	1 (50,0%)	27 (50,0%)	0,354	0,838
	Female	24 (49,0%)	2 (66,7%)	1 (50,0%)	27 (50,0%)		
Anatomical location	Mandible	48 (98,0%)	3 (100,0%)	2 (100,0%)	53 (98,1%)	0,104	0,949
	Maxilla	1 (2,0%)	0 (0,0%)	0 (0,0%)	1 (1,9%)		
	Anterior	5 (11,6%)	2 (66,7%)	0 (0,0%)	7 (14,6%)	8,013	0,091
Region*	Posterior	29 (67,4%)	1 (33,3%)	2 (100,0%)	32 (66,7%)		
	Anterior and Posterior	9 (20,9%)	0 (0,0%)	0 (0,0%)	9 (18,8%)		
Symptoms*	Absent	28 (82,4%)	3 (100,0%)	1 (50,0%)	32 (82,1%)	2,053	0,358
	Present	6 (17,6%)	0 (0,0%)	1 (50,0%)	7 (17,9%)		

* Information was not available in some cases of solid ameloblastoma.

Solid ameloblastomas affected patients with ages between 12 and 92 years (mean of 37.7 years). Desmoplastic ameloblastomas and hybrid lesions were respectively diagnosed in patients with ages between 20 and 51 years (mean of 34.3 years) and 44 and 71 years (mean of 57.5 years). In relation to size, solid ameloblastomas had diameters between 0.7 cm and 15.0 cm (mean of 4.3 cm), while desmoplastic ameloblastomas have a diameter between 3.0 cm and 4.0 cm (mean of 3.5 cm). Information regarding size was available in only one case (50.0%) of hybrid lesion, which showed a diameter of 5.0 cm. The time of evolution for solid lesions varied between 1 month and 33 years (mean of 36.9 months). Desmoplastic ameloblastomas and hybrid lesions showed, respectively, evolution times between 12 and 24 months (mean of 18 months) and 18 and 24 months (mean of 21 months).

**Figure 1:**
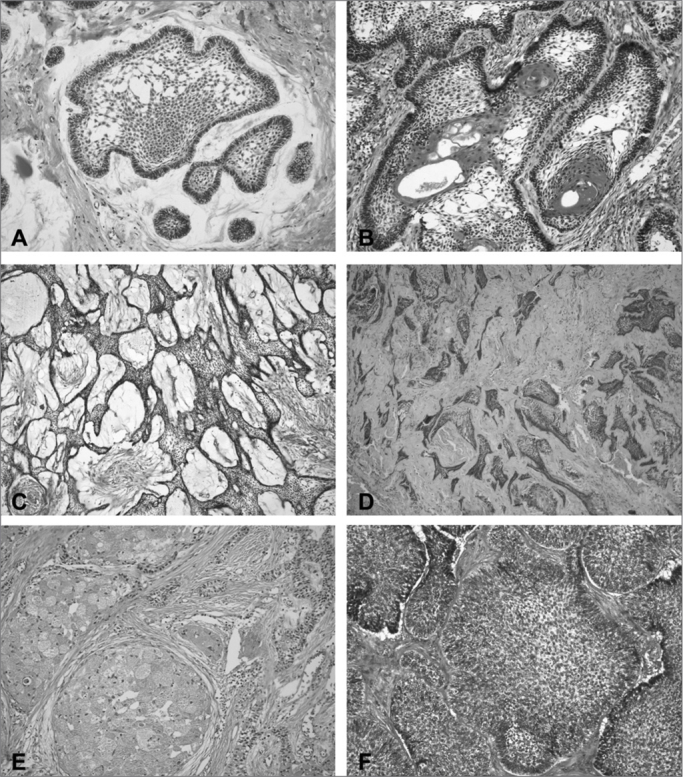
Microphotography of histological patterns. (A) Follicular pattern (H/E – 200x); (B) Acanthomatous pattern (H/E – 200x); (C) Plexiform pattern (H/E – 100x); (D) Desmoplastic pattern (H/E – 100x); (E) Granular cells pattern (H/E – 200x); (F) Basal cells pattern (H/E – 200x).

**Figure 2:**
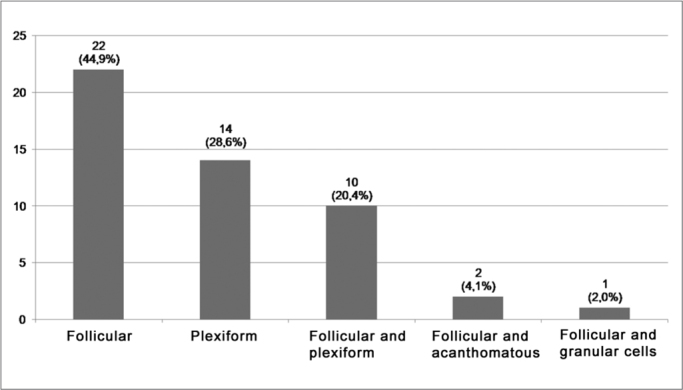
Frequency of the predominant histological pattern in solid ameloblastomas.

The follicular (77.6%) (Fig. 1A), acanthomatous (69.4%) (Fig. 1B) and plexiform (65.3%) (Fig. 1C) histologic patterns were the most frequent among the solid ameloblastomas. Notwithstanding, focal areas of desmoplastic ameloblastomas were identified in 11 solid lesions (22.4%) (Fig. 1D). And finally, granular cells and basal cells patterns were the least common in solid ameloblastomas, being identified in 12.2% (Fig. 1E) and 8.2% of the cases (Fig. 1F), respectively. The analysis of the predominant histological pattern in solid ameloblastomas revealed the follicular as the most frequent (44.9%), followed by the plexiform (28.6%) and the follicular/plexiform association (20.4%). The distribution of the cases of solid ameloblastoma in relation to the predominant histologic pattern is presented on Fig.2.

Among the hybrid lesions, the ratio of the solid ameloblastoma areas and that of desmoplastic ameloblastomas was similar (50.0%/50.0%). An assessment of solid ameloblastoma areas in the three hybrid lesions revealed only the histological patterns (100.0% of the cases), plexiform (100.0% of the cases) and acanthomatous (50.0% of the cases). Desmoplastic ameloblastomas had rare islets with a follicular pattern similar to those from solid lesions which were made almost exclusively of this histological pattern.

## DISCUSSION

Because of clinical and image differences between ameloblastomas made up exclusively of the desmoplastic pattern and solid lesions made up of the other histological patterns^4^, ^5^, ^6^, ^7^, ^8^, in the most recent classification of odontogenic tumors from the WHO^9^, the desmoplastic pattern was taken off the histological spectrum of solid ameloblastomas and fit within a distinct variant, called desmoplastic ameloblastoma.

Solid ameloblastomas affect the mandible prefferably^1^^,^^2^^,^^14^^,^^15^, especially the posterior region^1^^,^^15^, with a proportion between the gnathic bones of 1:5.4^16^. Our results corroborate the fact that these lesions have a predilection for the posterior mandible. Desmoplastic ameloblastomas affect predominantly the anterior maxillary bones^5^^,^^8^^,^^13^^,^^17^, ^18^, ^19^ and reveal a ratio varying between 1:0.6 and 1:1^6^^,^^9^^,^^18^. The findings of the present study agree with the preferential involvement of the anterior maxillary bones by desmoplastic lesions. Moreover, the reduced number of desmoplastic ameloblastomas seen in the present study corroborates the reports on the low frequency of this variant, encompassing between 0.5% and 13.0% of all ameloblastomas^1^^,^^2^^,^^4^^,^^5^^,^^13^^,^^15^^,^^18^^,^^19^.

Despite the aforementioned differences, solid and desmoplastic ameloblastomas share clinical traits. Both of them present asymptomatic and slow growth masses^1^^,^^9^^,^^18^^,^^19^ and, in general, are diagnosed in individuals between 30 and 60 years^1^^,^^4^^,^^9^^,^^11^. Moreover, solid and desmoplastic ameloblastomas have a relatively similar distribution between the genders^6^^,^^9^^,^^11^^,^^16^. The results from the present investigation are in agreement with these statements.

In sync with the similarities aforementioned, Keszler et al.^20^ carried out a comparative study with solid and desmoplastic ameloblastomas and did not report relevant differences between the genders, age or recurrence after treatment. For these authors, the desmoplastic ameloblastomas should not be considered a distinct clinicopathologic variant.

The size reported for the desmoplastic ameloblastomas varied between 1.0 cm and 8.5 cm^5^^,^^6^^,^^11^^,^^18^^,^^19^, usually with a diameter larger than 3.0 cm^4^, ^5^, ^6^^,^^17^^,^^21^. The average diameter for solid ameloblastomas varies between 4.3 cm^16^ and 6.2 cm^1^. Some cases may be very large, with diameters exceeding 15.0 cm^22^. In the present investigation, solid and desmoplastic lesions had a mean diameter of 4.3 cm and 3.5cm, respectively. Such findings match the literature data and stress the absence of significant differences between these variants as to the size of the lesions.

Duration time of the solid ameloblastomas can extend from 1 to 40 years^1^^,^^16^, with mean times between 27 months^16^ and 42.9 months^15^. Duration of the desmoplastic lesions can extend from 1 month all the way to 20 years^18^^,^^19^, with a mean time of 23 months^18^. In the present investigation, solid and desmoplastic lesions had a mean development time of 36.9 months and 18 months, respectively. These results match literature reports and there may be a relatively longer duration for desmoplastic ameloblastomas.

In the present study, the analysis of the predominant histological pattern in the solid lesions revealed the follicular (44.9%), the plexiform (28.6%) and the follicular/plexiform association (20.4%) as the most frequent ones. Similarly, Waldron and El-Mofty^13^ identified as predominantly common patterns in solid ameloblastomas the follicular (64.9%), the plexiform (16.9%) and the follicular/plexiform association (12.9%). Adebiyi et al.^2^ reported that most of the solid lesions belonged to the follicular type (70.4%), plexiform (14.1%) and acanthomatous (4.2%). Reichart et al.^16^ also reported the following solid lesions as being the most common patterns found: follicular (35.4%), plexiform (31.5%) and acanthomatous (11.8%). Our findings are in agreement with a greater frequency of follicular and plexiform patterns found in ameloblastomas.

In the present sample, most of the solid lesions (75.5%) were made up by more than one histological pattern. Similarly, in the study carried out by Adeline et al.^15^, 68.6% of the solid ameloblastomas revealed more than one histological pattern. Nonetheless, only 16.1% and 19.7% of the solid lesions evaluated by Reichart et al.^16^ and Kim and Jang^14^, respectively, showed more than one histological type. According to Adebiyi et al.^2^ and Waldron and El-Mofty^13^, ameloblastomas, especially the large ones, are made up of numerous histological patterns. For these authors, the quantity of tissue available for analysis has a relevant impact on the predominant histological type of ameloblastomas. Thus, it is possible that the differences seen between the present study and those by Reichart et al.^16^ and Kim and Jang14 are, in part, associated to the quantity of material available for microscopic evaluation.

In the present study, focal areas of desmoplastic ameloblastoma were seen in 11 (22.4%) solid lesions. Although the recent classification from the WHO^9^ describes the cases of solid and desmoplastic ameloblastomas as hybrid lesions, the rate of desmoplastic areas in these 11 solid lesions was not significant to classify them as hybrid. According to Waldron and El-Mofty^13^, the hybrid ameloblastoma lesions reveal typical areas of desmoplastic ameloblastomas in association with significant areas of solid ameloblastoma. Similarly, in the present study, the hybrid lesions revealed a similar ratio (50.0%/50.0%) among areas of solid and desmoplastic ameloblastomas. Thus, our studies reveal that focal areas of desmoplastic ameloblastomas can be identified with relative frequency in solid lesions.

Waldron and El-Mofty^13^ were the first to characterize the ameloblastoma hybrid lesion. Similarly to desmoplastic ameloblastomas, the hybrid lesions are rare, making up between 1.1%^10^ and 4.5% of all the ameloblastomas^13^. Cases have been seen in men and women between 25 and 82 years^10^^,^^13^^,^^17^^,^^23^^,^^24^. Most of the hybrid lesions affect the mandible^10^^,^^13^^,^^17^^,^^23^^,^^24^, and they can be restricted to the posterior region13 or simultaneously affect the anterior and posterior regions^10^^,^^17^^,^^24^. The results from the present investigation are in agreement with reports found in the literature.

In the hybrid lesions, the solid ameloblastoma areas are usually made up of the plexiform^13^ and follicular^10^^,^^23^ histological patterns. In the latter, sites of squamous metaplasia^10^^,^^23^ and keratinization^10^ have also been described.

Our findings are in agreement with these observations. Rarely, the solid areas of hybrid lesions show basal cells patterns^25^ or those of granular cells^10^.

Numerous aspects associated with ameloblastoma hybrid lesions are yet to be unveiled. We still do not know whether areas of desmoplastic ameloblastomas transform into solid ameloblastoma or desmoplastic alterations happen afterwards in the stroma of a solid ameloblastoma^11^. For Waldron and El-Mofty^13^, the absence of desmoplastic alterations seen in most of the solid lesions seen in their study would back up the first hypothesis.

According with Takata et al.^10^ and Philipsen et al.^11^, hybrid lesions could represent a coalition of tumors. Notwithstanding, Melrose^12^ states that the term “hybrid” does not have a clearly defined purpose and, if considered literally, can overestimate the meaning of seeing areas of desmoplastic ameloblastomas in combination with islets of solid ameloblastoma. The identification of focal areas of desmoplastic ameloblastoma in 22.4% of the solid lesions evaluated in this study suggest that it is not very likely that we would have a coalition of tumors and corroborate the statement from Melrose^12^.

We must stress that the low frequency of desmoplastic ameloblastomas and hybrid lesions makes it difficult to identify possible clinical differences between these lesions, as well as between them and solid ameloblastomas. In sync with this statement, the percentage differences between solid and desmoplastic ameloblastomas, seen in some clinical findings reported in the present study, did not have statistical significance. Thus, we stress the importance of carrying out multicentric studies which could assess a larger number of cases of desmoplastic ameloblastomas and hybrid lesions.

## CONCLUSION

The recent classification of odontogenic tumors of the WHO describes the desmoplastic ameloblastoma as a distinct variant and assigns the cases of coexistence between solid and desmoplastic ameloblastomas as hybrid lesions. Nonetheless, the results from this study reveal that focal areas of desmoplastic ameloblastomas can be identified with a relative frequency in solid ameloblastomas.
